# Data characterizing the genomic structure of the T cell receptor (TRB) locus in *Camelus dromedarius*

**DOI:** 10.1016/j.dib.2017.08.002

**Published:** 2017-08-03

**Authors:** Rachele Antonacci, Mariagrazia Bellini, Vito Castelli, Salvatrice Ciccarese, Serafina Massari

**Affiliations:** a Department of Biology, University “Aldo Moro” of Bari, Bari, Italy; b Department of Biological and Environmental Science e Technologies, University of Salento, Lecce, Italy

**Keywords:** T cell receptor, TRB locus, Dromedary genome, *Camelus dromedarius*, IMGT

## Abstract

These data are presented in support of structural and evolutionary analysis of the published article entitled “The occurrence of three D-J-C clusters within the dromedary TRB locus highlights a shared evolution in Tylopoda, Ruminantia and Suina” (Antonacci et al., 2017) [1]. Here we describe the genomic structure and the gene content of the T cell receptor beta chain (TRB) locus in *Camelus dromedarius.* As in the other species of mammals, the general genomic organization of the dromedary TRB locus consists of a pool of TRBV genes located upstream of in tandem TRBD-J-C clusters, followed by a TRBV gene with an inverted transcriptional orientation. A peculiarity of the dromedary TRB locus structure is the presence of three TRBD-J-C clusters, which is a common feature of sheep, cattle and pig sequences.

**Specifications Table**

**Table:** 

Subject area	*Biology, genetics, genomics*
More specific subject area	*Genetics, Genomics and Molecular Biology*
Type of data	*Tables and figures*
How data was acquired	*A standard BLAST search (Basic Local Alignment Search Tool.*http://blast.ncbi.nlm.nih.gov/Blast.cgi*.) of the public dromedary genomic assembly, Long PCR on genomic DNA and cloning*
Data format	*Analyzed*
Experimental factors	*Sequence analysis and dromedary DNA extraction*
Experimental features	*Dromedary lung genomic DNA was prepared from a single healthy animal. PCRs were performed by High Fidelity DNA polymerase. The PCR products were purified and cloned into the TA-vector system.*
Data source location	*Bari and Lecce, Italy*
Data accessibility	*The whole dromedary genome shotgun sequence is available at GenBank (ID: GCA_000767585.1). Sequence data published with this article were registered in EMBL database with the Accession number*LT837971

**Value of the data**

• These data insight into the genomic structure of the T cell receptor (TRB) locus in *Camelus dromedaries*. This results in the first, mostly complete, map of the TRB locus in a species of the Tylopoda suborder.
• The dromedary TRB locus characterization can be used to increase the understanding in the evolution of Camelidae and to contribute to solving the relative placement of this species within the Artiodactyla order.
• The availability of the sequence of the dromedary TRB locus allows researchers to concentrate on functional study and provides a tool to use this specie as a valuable model for immunological research.

## 1 Data

**Fig. 1:**
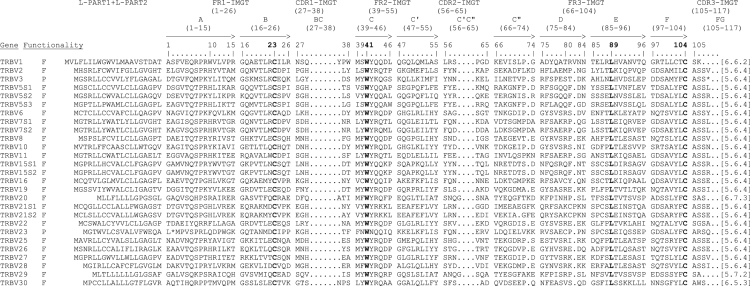
The IMGT Protein display of the dromedary TRBV genes. Only functional genes and in-frame pseudogenes are shown. The description of the strands and loops and of the FR-IMGT and CDR-IMGT is according to the IMGT unique numbering for V-REGION [6]. The amino acid length of the CDR-IMGT AA is also indicated in square brackets.

**Fig. 2:**
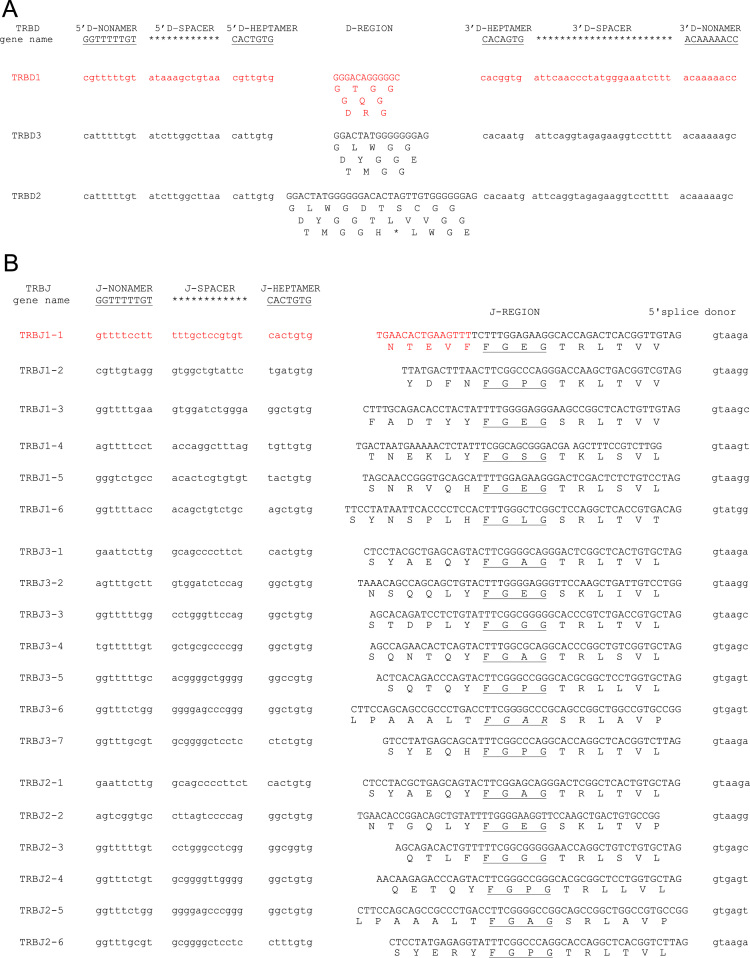

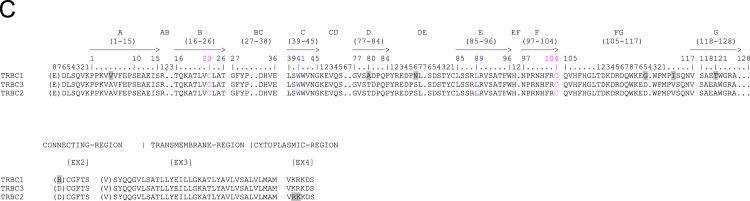
Nucleotide and deduced amino acid sequences of the dromedary TRBD (a), TRBJ (b) and TRDC (c) genes. The consensus sequence of the heptamer and nonamer are provided at the top of the figure and underlined. The numbering adopted for the gene classification is reported on the left of each gene. The gene sequence retrieved from the Ca_dromedarius_V1.0 genomic assembly is highlighted in red. In (a), the inferred amino acid sequence of the TRBD genes in the three coding frames are reported. In (b), the donor splice site for each TRBJ is shown. The canonical FGXG amino acid motifs are underlined. The unusual TRBJ3.6 gene motif is in italics. In (c), IMGT Protein display of the dromedary TRBC genes. Descriptions of the strands and loops were collected according to the IMGT unique numbering for C-DOMAIN [7].

Data presented in the text include tables and figures giving information on the genomic structure and the gene content of the dromedary TRB locus, a mammalian species belonging to the *Camelus* genus. This information was obtained by integrating the sequence data deduced from the public genomic assembly [2] with sequences obtained by PCR experiments conducted in our laboratory. Table 1 describes position, classification and functionality of the TRB genes retrieved from the dromedary public genome assembly. Table 2 shows the description of the dromedary TRBV pseudogenes. Table 3 describes position, classification and functionality of the unrelated TRB genes recovered from the dromedary public genome assembly. Fig. 1 shows the deduced amino acid sequences of the dromedary TRBV genes according to IMGT unique numbering for the V-REGION [6]. Table 4 provides the list of the genomic clones of the dromedary TRBD-J-C region with the primer pairs used and the PCR conditions. Fig. 2 shows the TRBD, the TRBJ and the TRBC gene sequences.

**Table 1:** Description of the TRB genes in the *Camelus dromedarius* genome assembly. The position of all genes and their classification and functionality are reported.

**Gene classification**	**Functionality**^a^	**NCBI Reference Sequence**	**Position**^b^
TRBV1	F	NW_011591622	861263-861886
TRBV2	F	NW_011591622	932263-932714
TRBV3	P	NW_011591622	927952-928412
TRBV5S1	F	NW_011591622	937384-937843
TRBV5S2	F	NW_011591622	940879-941358
TRBV5S3	F	NW_011591622	955293-955748
TRBV6	F	NW_011591622	944809-945237
TRBV7S1	F	NW_011591622	947134-947581
TRBV7S2	F	NW_011591622	962228-962689
TRBV8	F	NW_011591622	950124-950593
TRBV9	P	NW_011591622	965923-966346
TRBV10	F	NW_011591622	970368-970809
TRBV11	F	NW_011591622	975860-976308
TRBV12S1	P	NW_011591622	981727-982197
TRBV12S2	P	NW_011591622	992125-992569
TRBV14	P	NW_011591622	995472-995906
TRBV15S1	F	NW_011591622	997569-998023
TRBV15S2	F	NW_011591622	999129-999583
TRBV16	F	NW_011591622	1003645-1004098
TRBV19	F	NW_011591622	1018094-1018641
TRBV20	F	NW_011591622	1020910-1021565
TRBV21S1	F	NW_011591622	1028337-1028797
TRBV21S2	F	NW_011591151	70843-70731
TRBV21S3	P	NW_011591151	62738-62511
TRBV22	F	NW_011591151	46518-46381
TRBV23	P	NW_011591151	60590-60480
TRBV24	P	NW_011591151	56428-56106
TRBV25	F	NW_011591151	52347-52219
TRBV26	F	NW_011591151	66428-66297
TRBV27	F	NW_011591151	41158-41032
TRBV28	F	NW_011591151	32762-32640
TRBV29	F	NW_011591151	27109-26837
TRBD1	F	NW_011591151	9932-9943
TRBJ1-1	F	NW_011591151	9247-9294
TRBJ1-2	F	NW_011591151	9116-9159
TRBJ1-3	F	NW_011591151	8861-8910
TRBJ1-4	F	NW_011591151	8258-8308
TRBJ1-5	F	NW_011591151	7982-8031
TRBJ1-6	F	NW_011591151	7491-7543
TRBC1	F	NW_011591151	EX1 4773-5166
			EX2 4311-4328
			EX3 4044-4150
			EX4 3711-3731
TRBC3	nd	NW_011591151	EX2 2866-2883
			EX3 2599-2705
			EX4 2266-2286
TRBJ3-1	F	NW_011620189	653-702
TRBJ3-1	F	NW_011601111	2234-2283
TRBJ3-2	F	NW_011601111	2426-2476
TRBJ3-3	F	NW_011601111	2642-2690
TRBJ3-4	nd	NW_011601111	2787-2814
TRBJ2-2	F	NW_011616084	215-265
TRBJ2-3	nd	NW_011616084	2-46
TRBJ2-6	nd	NW_011607149	185-231
TRBC2	nd	NW_011593440	EX1 1911-2149
			EX2 2622-2639
			EX3 2800-2906
			EX4 3190-3210
TRBV30	F	NW_011593440	14509-14160

a nd: not defined (indicates that the nt sequence of the gene is incomplete and its functionality cannot be defined).

b L-PART1/ V-exon for TRBV genes and coding sequence for TRBD and TRBJ.

**Table 2:** Description of the *Camdro* TRBV pseudogenes.

**TRBV genes**	**Defective Leader**	**Frameshift**	**Stop codon**	**Defective splice sites**	**Defective RSS**
TRBV3			●		
TRBV9	●				●
TRBV12S1		●			
TRBV12S2		●			
TRBV14		●			
TRBV21S3	●	●			
TRBV23			●		
TRBV24		●		●

**Table 3:** Description of the unrelated TRB genes in the *Camelus dromedarius* genome assembly. The position of all genes and their classification and functionality are reported.

**Gene classification**	**Functionality**^a^	**NCBI reference sequence**	**Position**
MOXD2	F	NW_011591622	850155-856730
TRY1	F	NW_011591622	870036-876394
TRY2	F	NW_011591622	882909-888072
TRY3	nd	NW_011623391	1-2387
TRY4	F	NW_011591151	13974-17714
EPBH6	F	NW_011593440	46466-60647

a nd: not defined (indicates that the nt sequence of the gene is incomplete and its functionality cannot be defined).

**Table 4:** *Camelus dromedarius* D-J-C region genomic clones. The primer sequences, the PCR conditions and the size of each clone are reported.

Clone	Primer pairs sequence (5′-3′)	Primer location	T annealing	Product length (bp)
pSCBJ11	JB11U: CTTTGGAGAAGGCACCAG	TRBJ1-1 gene	55/58	4396
	CB2L: TGGTTGCGGGGGTTGTGC	TRBC gene exon 1		
pSCJ22KN	CB2U: GCACAACCCCCGCAACCA	TRBC gene exon 1	53/55	5000
	JB34L: GCCAAAGTACTGAGTGTT	TRBJ3-4 gene		
pSCBJ27U	JB34U: AACACTCAGTACTTTGGC	TRBJ3-4 gene	56/58	4077
	CB2L: TGGTTGCGGGGGTTGTGC	TRBC gene exon 1		
pSCBD3	CB2U: GCACAACCCCCGCAACCA	TRBC gene exon 1	55/56	4848
	JB23L: CCGCCGAAAAACAGTGTC	TRBJ2-3 gene		
pSCMG1	JB23U: GACACTGTTTTTCGGCGG	TRBJ2-3 gene	55/58	3160
	CB2L: TGGTTGCGGGGGTTGTGC	TRBC gene exon 1		
pSCB2C8	CB2U: GCACAACCCCCGCAACCA	TRBC gene exon 1	62	1331
	3UTR:GTTGAGCTCACTTTGCAGGG	TRBC2 gene 3UTR		

## 2 Experimental design, materials and methods

### 2.1 Analysis of the dromedary TRB locus retrieved from the genome assembly: identification of the related and unrelated TRB genes

We employed the recent submission to NCBI (BioProject PRJNA234474) of a draft genome sequence from the Arabian camel [2] to identify the TRB locus in this species. A standard BLAST search (Basic Local Alignment Search Tool. http://blast.ncbi.nlm.nih.gov/Blast.cgi.) of the dromedary genomic resource was then performed by using human and sheep TRB gene sequences to assess their physical location in the dromedary genome. We directly retrieved a sequence of 457871 pb (gaps included) from the PRJNA234474_Ca_dromedarius_V1.0 assembly that corresponds to eight distinct unplaced and not continuous scaffolds (Fig. 1 in [1]). The sequence comprises the MOXD2 and the EPHB6 genes that flank the 5′ and 3′ ends, respectively, of all mammalian TRB loci studied to date. All dromedary TRB genes have been recognized and annotated while taking into account both the human sequence and the sheep genomic D-J-C region as a reference [3], [4], [5] (Table 1). The functionality of V, J and C genes was predicted through the manual alignment of sequences adopting the following parameters: (a) identification of the leader sequence at the 5′ of the TRBV genes; (b) determination of proper recombination signal (RS) sequences located at 3′ of the TRBV, 5′ of the TRBJ, and 3′ and 5′ ends of the TRBD genes, respectively; (c) determination of correct acceptor and donor splicing sites; (d) estimation of the expected length of the coding regions; (e) absence of frameshifts and stop signals in the coding regions of the genes. We annotated 33 TRBV germline genes (twenty-five functional genes and eight pseudogenes) (Table 2), one TRBD, 13 TRBJ and two complete and one incomplete TRBC genes. The analysis of the 3′ part of the locus revealed the potential presence of three D-J-C clusters similar to clusters found in sheep [4], [5].

We also identified and annotated four trypsin-like serine protease (TRY) genes (Table 3). In this context, downstream of the TRBV1 gene, proceeding from 5′ to 3′, we found as in humans two protease genes that we recognized tentatively, according to their genomic position, as TRY1 (alias PRSS58 or TRYX3) and TRY2 (alias TRY2P), respectively. A third TRY gene, named TRY3, was homologous to a gene located after the TRY2P gene in humans that was found within the NW_011623391 unplaced scaffold. Extrapolation of the synteny with the human sequence predicts that the NW_011623391 scaffold should be juxtaposed within the dromedary TRB locus, upstream of the TRBV3 gene (Fig. 1 in [1]). An additional TRY gene, classified as TRY4, was found before the D-J-C region. Thus, unlike humans, only one TRY gene encompasses the array of the TRBV genes. All dromedary TRY genes appear putatively functional with the presence of correct acceptor and donor splicing site and an absence of frameshifts and stop codon in their coding regions. The genomic structure of the MOXD2 and EPHB6 genes, which delimit the TRB locus, was also defined (Table 3).

### 2.2 Protein display of the dromedary TRBV genes

The deduced amino acid sequences of the germline TRBV genes were manually aligned according to IMGT unique numbering for the V-REGION [6] to maximize the percentage of identity (Fig. 1). Only potential functional genes and in-frame pseudogenes are shown. All sequences exhibit the typical framework regions (FR) and complementarity determining regions (CDR) as well as the four amino acids: cysteine 23 (1st-CYS) in FR1-IMGT, tryptophan 41 (CONSERVED-TRP) in FR2-IMGT, hydrophobic amino acid 89, and cysteine 104 (2nd-CYS) in FR3-IMGT [6]. Conversely, CDR-IMGT varies in amino acid composition and length. It should be noted that the TRBV21 genes show a difference in length of one amino acid in the FR3 that corresponds to a C′′ strand that is shorter and has a diverse amino acid sequence for TRBV21S2 compared to the TRBV21S1 gene.

### 2.3 Isolation of the dromedary TRBD-J-C region and analysis of the gene content

To isolate the entire TRBD-J-C region, we set up six different PCRs to produce six consecutive amplicons that cover the region between the first TRBJ and the last TRBC gene. Mostly, for each amplification, we used a primer pair, a gene-specific primer designed on the sequence of the TRBJ genes identified within the cDNA clones (see [1]), and a conserved primer constructed on the first exon of the TRBC genes. For the isolation of the TRBC2 gene, a 3'UTR lower primer derived from the sequence of the genomic assembly was used. Amplification consisted of an initial denaturation step at 93 °C for 2 min followed by 10 amplification cycles that each comprised a denaturation step at 93 °C for 10 s, an annealing step with a low temperature (53–56 °C, according to the melting temperature of the primers) for 30 s, an extension step at 68 °C for 7 min, followed by 25 cycles with a higher annealing temperature (55–58 °C, according to the melting temperature of the primers) and a gradually increasing extension time of 20 s as well as a final incubation at 68 °C for 7 min. A 30-deoxyadenosine overhang was added to blunt-ended amplicons by incubation with 1.0 unit of Platinum Taq DNA Polymerase (Invitrogen) at 72 °C for 10 min. These products were purified and cloned into the StrataClone TA-vector per the manufacturer's instructions. For each sample, 6 to 10 colonies were propagated and bi-directionally sequenced using M13 and T7 vector-specific primers. All plasmid sequence data were manually analysed. For the list of the clones with the primer pairs used and the PCR conditions see Table 4. All the obtained amplicons were sequenced (Acc. no. LT837971). The sequenced region is schematically illustrated in Fig. 3 in [1].

The nucleotide and deduced amino acid sequences of the TRBD, TRBJ and TRBC genes classified according to the similarity to the sheep sequence are shown in Fig. 2.
