# Supplementary material for: Data characterizing the genomic structure of the T cell receptor (TRB) locus in Camelus dromedarius
Source: Data Brief. 2017 Aug 3;14:507–14. doi: 10.1016/j.dib.2017.08.002 (PMC5562110; doi:10.1016/j.dib.2017.08.002)
Supplement: Supplementary file 1 — Transparency document [file mmc1.docx]

**Conflict of interest:** The authors declare no financial or commercial conflict of interest.
